# Association of markers of chronic viral hepatitis and blood mercury levels in US reproductive-age women from NHANES 2001–2008: a cross-sectional study

**DOI:** 10.1186/1476-069X-11-62

**Published:** 2012-09-12

**Authors:** Mary C Sheehan, Thomas A Burke, Patrick N Breysse, Ana Navas-Acien, John McGready, Mary A Fox

**Affiliations:** 1 Department of Health Policy and Management, Johns Hopkins Bloomberg School of Public Health, Baltimore, MD, USA; 2 Department of Environmental Health Sciences, Johns Hopkins Bloomberg School of Public Health, Baltimore, MD, USA; 3 Department of Statistics, Johns Hopkins Bloomberg School of Public Health, Baltimore, MD, USA

**Keywords:** Biomonitoring, Developmental neurotoxicity, Hepatitis, Mercury, NHANES, Reproductive-age women, Seafood, Susceptibility

## Abstract

**Background:**

Methylmercury (MeHg) is a neurotoxin primarily found in seafood; exposures in reproductive-age women are of concern due to vulnerability of the developing fetus. MeHg is mainly eliminated via an enterohepatic cycle involving the liver and gallbladder. Dysfunction in these organs has been associated with slower MeHg elimination in laboratory animals. We hypothesized that women testing positive for chronic hepatitis B (HBV) or C (HCV), both associated with risk of longer-term liver and gallbladder impairment, would have higher total blood mercury (TBHg) concentrations than those negative for the viruses, reflecting slower MeHg elimination.

**Methods:**

Geometric mean (GM) TBHg levels from a representative sample of over 5,000 seafood-consuming, reproductive-age women from eight years (2001–2008) of the US NHANES survey were compared by viral hepatitis status (as determined by serological assay) using multiple linear regression. Adjustment was made for estimated MeHg intake from seafood consumption, social and demographic variables and other predictors.

**Results:**

Women with chronic HBV had 1.52 (95% CI 1.13, 2.05, p < 0.01) times the GM TBHg of women who had not come into contact with the virus. The positive association was strongest in those with most severe disease. A modest negative association was found with HCV markers.

**Conclusions:**

While study design prevents inferences on causality, the finding that MeHg biomarkers differ by hepatitis status in this population suggests viral hepatitis may alter the pace of MeHg elimination. Offspring of HBV-infected seafood-consuming women may be at higher risk of MeHg-induced developmental delays than offspring of those uninfected. Possible reasons for the unanticipated negative association with HCV are explored.

## Background

Methylmercury (MeHg) is a neurotoxin widely present in the environment to which the developing fetus is acutely vulnerable [1]. The primary exposure source and pathway for this sensitive population is maternal seafood consumption [2]. Numerous countries have issued commercial seafood advisories for reproductive-age women [3] with the goal of minimizing MeHg intake and protecting offspring from the verbal, spatial and fine-motor skill delays associated with MeHg-induced developmental neurotoxicity [2].

Individuals are known to differ in susceptibility to health risks from contaminants such as MeHg [4,5]. Variability has been observed in MeHg’s toxicokinetic processing as well as its neurotoxic effects in association with differences in maternal age [6], genetic polymorphisms in the glutathione (GSH) system [7], nutrient intake [8,9] and the social environment [10], among other factors. Further study of such effect modifiers has been called for [11,12]. In particular, while substantial variability has been demonstrated in MeHg’s elimination phase [1,13], few epidemiological studies have examined factors that modify the pace of elimination. Infants born to seafood-consuming reproductive-age women who are slow eliminators of MeHg may be at higher risk of neurological harm.

MeHg is eliminated primarily (more than two-thirds) via the biliary pathway [14]. Animal studies have shown that elimination of MeHg, like many nutrients, drugs and xenobiotics, involves an enterohepatic (EH) cycle [15]. According to Dutczak et al. [16]:

“Reabsorption of solutes from bile… functions to conserve endogenous compounds but can also prove deleterious by impeding the clearance of toxic compounds… MeHg … [is] believed to undergo extensive enterohepatic cycling… [in which] the gallbladder … reabsorbs MeHg reducing the amount delivered to the GI tract, and therefore the amount that can be eliminated…”

EH reabsorption of MeHg helps explain its relatively long half-life (averaging about 50–70 days) in the human body [15,17]. Factors such as prescription drugs, age, and disease have been observed to speed or slow the pace of EH cycling [18,19]. In particular, it may be hypothesized that dysfunction in the liver and gallbladder, key organs in the EH cycle, may contribute to the wide variability in MeHg half-life observed (from 32 to 189 days) [17]. Experimental research has found that depletion of GSH, which can occur when the liver functions poorly, decreased biliary excretion of MeHg and increased its half-life in male rats [20]. In biliary excretion-deficient male rats lower bile flow was associated with a 2.5-times longer MeHg elimination time than in control animals [21]. In humans, liver that is damaged by inflammation, fibrosis (hardened, fibrous tissue) or cirrhosis (replacement of healthy by scar tissue) has a lower share of normally-functioning tissue than healthy liver [22]; blood flow and metabolism are reduced, metabolic byproducts may be less efficiently transported into bile, and bile less efficiently cleared [22,23]. Such conditions may affect hepatobiliary clearance by decreasing uptake of substances into hepatocytes, changing metabolism in hepatocytes, or decreasing elimination into bile [18].

The most common cause of liver disease is viral hepatitis [24,25]. In the US, 4.9% of the population has been exposed to hepatitis B (HBV) and the prevalence of chronic HBV is 0.3%; hepatitis C (HCV) exposure is estimated to be 1.8% and prevalence of chronic disease is 1.6% [25]. The common general characteristics of both chronic HBV and HCV include premature death of hepatocytes, inflammation, and fibrosis [26]. Up to 30% of HBV carriers and 65% of the HCV-infected go on to develop cirrhosis (the highest stage of fibrosis) and/or hepatocellular carcinoma (HCC) [24,27]. Chronic hepatitis can also lead to structural changes in biliary elimination architecture [18,27,28]. Specifically, viral hepatitis has been associated with thickening of the gallbladder wall and development of gallbladder sludge [29], presence of gallstones [30], and gallbladder and bile duct polyps and cancers [31,32] in epidemiological studies in varied populations. Bile acids, which also undergo EH cycling, have been found to be elevated in serum in cases of hepatitis-associated liver dysfunction [27].

Based on this literature, we hypothesized that chronic viral HBV or HCV could interfere with biliary elimination of MeHg, and that this would be reflected in higher circulating blood levels of mercury in those with these diseases compared to those without. As reference scenarios, we further hypothesized no difference in blood mercury concentrations between those with and those without the acute-only and self-limiting hepatitis A (HAV), and between those vaccinated and those unvaccinated for HBV.

## Methods

### Study population and data

We tested this hypothesis in a large sample of reproductive-age seafood-consuming women who participated in the National Health and Nutrition Examination Survey (NHANES). NHANES is a complex, stratified multi-stage sample designed to be representative of the US non-institutional population, implemented by the CDC’s National Center for Health Statistics (NCHS). Data collection consists of an in-person interview to gather demographic, socioeconomic and other information, and a physical examination. The exam includes a dietary questionnaire and laboratory testing of blood, urine and other tissue samples for a standard biochemical and disease panel and numerous chemical toxicants. In order to have a sufficient sample size to stratify by gender, lifestage, seafood intake, and hepatitis status, we pooled data from 2001 to 2008, the most recent four NHANES cycles with full data available. The total screened sample for the eight-year period was 50,822; the average response rate for the interview was 80%, and for the exam was 77%. We analyzed data for 7,870 women aged 16–49 who completed the NHANES questionnaire, examination and laboratory components. We eliminated women who answered negatively to having consumed seafood in the last 30 days (as these women were less likely to have detectable blood mercury concentrations), as well as those missing blood mercury measurements and other covariates of interest. The total number of study participants was 5,088 for the HBV analysis; 5,071 for HCV; and 4,104 for HAV (HAV assay results were available for 2001–2006 only). Informed-consent was provided by all participants, and the survey protocol was approved by the NCHS institutional review board.

### Blood mercury measurement

The main dependent variable was the concentration of total blood mercury (TBHg), a validated proxy measure for recent MeHg intake [33,34]. TBHg was measured in micrograms per liter (μg/L) of whole blood using cold vapor atomic absorption spectrometry (2001–2002) and inductively-coupled plasma dynamic reaction cell mass spectrometry (2003–2008). Analysis of samples was done at the CDC’s Division of Laboratory Sciences, National Center for Environmental Health (NCEH). All medical and phlebotomy staff were trained and used standardized protocols. Other than measurement technique, no changes in lab procedures were noted over the study period. Quality assurance included use of blind split samples, repeat testing and use of international standard rules for quality control. NCEH staff verified extremely high and low values and performed consistency checks. The limit of detection was between 0.14 μg/L and 0.33 μg/L.

### Determination of hepatitis status and study hypotheses

The main independent variable for this analysis was hepatitis status. Hepatitis status was determined using CDC case definitions [35,36], the hepatitis literature, and hepatitis assay results for our 2001–2008 sample of reproductive-age women (Table 1). For the women in this NHANES sample, laboratory results analyzed by the CDC’s Division of Viral Hepatitis were available for a sub-set of the assays noted in the CDC chronic HBV and HCV case definitions: hepatitis B core antibody (anti-HBc); hepatitis B surface antibody (anti-HBs); hepatitis B surface antigen (HBsAg), and hepatitis C antibody (anti-HCV). Results were also available for total hepatitis A antibody (anti-HAVT). We examined three models.

**Table 1:** Hepatitis groups, available NHANES test results and number of women in study sample

**Hepatitis group**	**NHANES tests available and results to be in group**	**No. women in sample**
**Hepatitis A**		
Current or past infection or immunization	Anti-HAVT positive	1,466
No infection or immunization	Anti-HAVT negative	2,638
**Hepatitis B**		
Current infection	Anti-HBc positive, anti-HBs negative	40
*- Active infection*	*As for current infection, plus HBsAg positive*	*11*
*- Inactive carrier*	*As for current infection, plus HBsAg negative*	*29*
Resolved (past) infection	HBsAg negative, anti-HBc positive, anti-HBs positive	145
Immunized	HBsAg negative, anti-HBc negative, anti-HBs positive	1,520
No infection or immunization	HBsAg negative, anti-HBc negative, anti-HBs negative	3,383
**Hepatitis C**		
Current or past infection	Anti-HCV positive	70
No infection	Anti-HCV negative	5,001

In the Hepatitis A model, comparison was made between women who tested positive for total hepatitis A antibody (anti-HAVT) – which indicates current or past exposure to either the HAV virus or the vaccine [24] – and the reference (negative anti-HAVT) group. Anti-HAVT was determined using a solid-phase competitive enzyme-based immunosorbent assay (ELISA). Because the acute-only HAV infection carries low risk of longer-term liver damage, in this model we hypothesized there would be no association between TBHg concentrations and positive anti-HAVT status.

HBV has a more complex natural history, with four recognized stages of disease: virus incubation, immune response, recovery (inactive carrier status), and development of immunity; while an average of 1-2% of inactive carriers seroconvert and develop natural immunity annually, about one-third may at some point re-activate to active disease [24,26]. Positive anti-HBc accompanied by positive HBsAg indicates chronic active disease, while in combination with negative HBsAg it indicates likely inactive carrier status. Anti-HBs is a marker of immunity; in combination with anti-HBc this is likely to be natural immunity, while anti-HBs positive alone signals probable vaccine-derived immunity [24,26,37]. Based on these serological assays we defined three HBV-exposed groups:

• Current HBV: women positive for anti-HBc and negative for anti-HBs, including both those HBsAg positive (likely to have active disease) and those HBsAg negative (likely to be inactive carriers);

• Recovered HBV: women positive for both anti-HBc and anti-HBs, denoting past infection and development of natural immunity to the virus; and

• Immunized HBV: women positive for anti-HBs and negative for anti-HBc, indicating vaccine-derived immunity.

Each HBV-exposed group was compared to the reference group negative for anti-HBc, anti-HBs and HBsAg (i.e., no contact with the virus or vaccine). Anti-HBc was detected using an ELISA (Ortho) in which all reactive specimens were re-tested (the result was positive with at least one reactive re-test). Only positive anti-HBc samples were tested for HBsAg, detected using a sandwich ELISA (Abbott Labs). Anti-HBs was determined using a sandwich solid phase ELISA (AUSAB), with concentration of anti-HBs compared to a standard curve. Because of the elevated risk of longer-term liver and gallbladder damage we hypothesized a positive association between presence of markers for current HBV and TBHg levels. Since the seroconverted remain at elevated risk of developing fibrosis, cirrhosis and HCC over their lifetimes [24], we also hypothesized an association between markers of HBV recovery and TBHg levels. We hypothesized no association between having been vaccinated for HBV and TBHg.

In model C, we compared women testing positive for anti-HCV, indicative of current or past infection [24,26], and the reference uninfected group. Anti-HCV was detected using direct solid-phase ELISA screening, and positives confirmed with recombinant immunoblot assay (Chiron). Due to longer-term liver damage associated with HCV, we hypothesized a positive association between presence of anti-HCV and TBHg concentrations. There is currently no vaccine for HCV.

### MeHg intake and other covariates

Seafood consumption frequency and species are the main determinants of MeHg intake in the non-occupational general human population [1,2,34]. The NHANES dietary interview covered consumption frequency of 10 shellfish and 21 finfish species (number of servings over the 30 days prior to the interview). Trained interviewers used a computerized data collection instrument (involving a five-step recall process: quick list, forgotten foods prompt, time and occasion prompt, details prompt, and final probe), along with aids such as photos of different seafood species. Quality control included re-confirming all values over nine servings and review of interviewer audio reports, among other measures. We used these seafood consumption data, along with seafood species-specific mercury concentration data published by the US FDA [38], to calculate estimated MeHg intake by study participant. MeHg intake for the six most frequently-consumed species was estimated by multiplying the number of meals for each over 30 days by per-meal mercury values in grams, derived using the FDA average MeHg concentration for the species (assuming a 170-gram meal size [39]). To estimate MeHg intake from less commonly-consumed other finfish and shellfish we used average FDA MeHg concentration values for all finfish and all shellfish, respectively. The results were aggregated by participant and used to adjust the three models for 30-day estimated MeHg intake from seafood.

Additional independent variables that could potentially confound results were defined based on the mercury literature [34,40-42]. These included household income, education attainment, ethnicity, country of birth, alcohol intake, and body-mass index (BMI). We adjusted each model for other hepatitis viruses, and given co-infection with human immunodeficiency virus (HIV) among some hepatitis-infected groups [43], we also included HIV status as a covariate.

### Statistical methods

To facilitate use of linear regression methods, we log-transformed the right-skewed TBHg distribution. We employed the back-transformed geometric mean (GM) as a summary statistic, and used 95% confidence intervals (95% CIs) to describe variation. Using bivariate analysis, we summarized GM TBHg concentrations by covariate, and participant characteristics by hepatitis status. We used linear regression analysis to examine relationships between TBHg and hepatitis status defined by the serological markers described. Regression coefficients were back-transformed from the log scale. Our main outcome was the ratio of GM TBHg concentration between women in the defined hepatitis-exposed group to the respective unexposed reference group. A GM TBHg ratio of greater than 1.0 indicated a positive association, and a GM TBHg ratio of less than 1.0 indicated a negative association. Statistical significance was tested using a two-tailed *t*-test with an alpha level of 0.05. We adjusted each model for the covariates described using backwards step-wise selection to eliminate covariates (p-value cut-off of less than 0.10). In addition, we examined interaction terms to test for effect modification, and performed sensitivity analyses to examine assumptions about seafood intake, the most important driver of TBHg levels. In sensitivity analysis we also examined clinical markers of liver function. We weight-adjusted all analyses to account for over-sampling of under-represented groups, and used Taylor series linearization for variance estimation as recommended by NCHS. All statistical analyses were performed in Stata (Stata Statistical Software: Release 10, Statacorp, College Station, TX, 2007).

## Results

### Blood mercury concentrations

The GM TBHg concentration in this population of reproductive-age seafood-consuming women was 1.02 (95% CI 0.97, 1.08) μg/L (Table 2). Consistent with the literature [34,40,42], unadjusted GM TBHg concentrations were higher in older women, those of “Other” (Asian, Pacific Islander and Native American) ethnicity, the foreign-born, those with higher household income and education attainment, and those with lower BMI. Women who consumed 5–8 servings of seafood over the month had GM TBHg concentrations almost two times higher than those who consumed 1–4 servings. Those who consumed 9 or more servings had GM TBHg concentrations over two times higher than the 1–4 serving group. There was a moderate correlation between TBHg and seafood servings (correlation coefficient 0.34). Compared to women without exposure to a hepatitis virus, those positive for HAV had similar GM TBHg concentrations. Those positive for HBV had 1.4 times higher GM TBHg concentrations, and those positive for HCV had 25% lower GM TBHg concentrations, than those without exposure to a hepatitis virus.

**Table 2:** Total blood mercury concentrations (μg/L) in study participants by selected covariates

	**n =** ^1^	**GM**	**95% CI**	**5**^**th **^**P**	**25**^**th **^**P**	**50**^**th **^**P**	**75**^**th **^**P**	**95**^**th **^**P**
**Total**	5,189	1.02	(0.97, 1.08)	0.20	0.58	1.00	1.87	4.82
**Age** (yrs)								
- 16 – 19	1,103	0.74	(0.67, 0.82)	0.20	0.40	0.77	1.40	3.10
- 20 – 29	1,404	0.88	(0.81, 0.94)	0.20	0.50	0.88	1.66	3.70
- 30 – 39	1,358	1.13	(1.02, 1.24)	0.20	0.60	1.10	2.20	5.80
- 40 – 49	1,324	1.14	(1.07, 1.22)	0.28	0.70	1.10	1.90	5.00
**Ethnicity**								
- White	2,100	1.00	(0.92, 1.07)	0.20	0.53	1.00	1.85	4.89
- Mexican American	1,248	0.77	(0.71, 0.84)	0.20	0.49	0.80	1.30	2.91
- Other Hispanic	328	1.04	(0.84, 1.29)	0.20	0.66	1.09	1.90	3.59
- Black	1,285	1.08	(0.99, 1.17)	0.30	0.63	1.04	1.75	4.10
- Other	228	1.90	(1.62, 2.24)	0.40	0.90	2.03	3.60	8.50
**Country born**								
- US	4,008	0.98	(0.92, 1.04)	0.20	0.54	0.95	1.70	4.45
- Foreign	1,180	1.28	(1.15, 1.42)	0.28	0.70	1.25	2.49	6.52
**Household income** (/yr)								
- < $45,000	2,580	0.86	(0.80, 0.92)	0.20	0.50	0.82	1.54	3.64
- $45,000-$75,000	1,070	1.03	(0.95, 1.11)	0.23	0.60	1.00	1.80	4.90
- > $75,000	1,217	1.29	(1.19, 1.30)	0.30	0.60	1.27	2.30	5.82
**Education**								
- < HS diploma	1,695	0.76	(0.70, 0.83)	0.20	0.40	0.75	1.39	3.30
- HS diploma	1,052	0.93	(0.85, 1.00)	0.20	0.50	0.90	1.60	4.01
- BA degree	1,496	1.02	(0.94, 1.11)	0.20	0.60	1.00	1.80	4.10
- > BA degree	944	1.37	(1.26, 1.49)	0.30	0.72	1.35	2.62	6.40
**BMI** (kg/m^2^)								
- < 30	3,920	1.10	(1.03, 1.17)	0.20	0.60	1.10	2.00	5.16
- > = 30	1,706	0.89	(0.83, 0.95)	0.20	0.50	0.86	1.50	4.00
**Alcohol** (g/day)								
- < 10	4,500	0.97	(0.91, 1.03)	0.20	0.53	0.93	1.71	4.50
- > = 10	683	1.29	(1.18, 1.42)	0.30	0.71	1.29	2.31	5.58
**Seafood** (/mo.)								
- 1–4 servings	4,422	0.93	(0.88, 0.99)	0.20	0.52	0.90	1.62	4.17
- 5–8 servings	458	1.72	(1.55, 1.91)	0.41	0.90	1.76	3.01	7.52
- >/= 9 servings	198	2.08	(1.81, 2.39)	0.65	1.14	1.92	3.70	10.60
**Hepatitis A**								
- No infection or immunization	2,669	1.03	(0.99, 1.07)	0.20	0.60	1.00	1.90	4.90
- Infection or immunization	1,484	1.04	(0.99, 1.05)	0.23	0.60	1.00	1.90	5.10
**Hepatitis B**								
- No infection or immunization	4,957	1.01	(0.96, 1.07)	0.20	0.57	1.00	1.81	4.70
- Past or current infection ^2^	185	1.39	(1.16, 1.65)	0.20	0.70	1.30	3.80	6.90
- Immunization	1,673	1.08	(1.03, 1.13)	0.20	0.68	1.07	1.98	4.90
**Hepatitis C**								
- No infection	5,055	1.03	(0.97, 1.09)	0.20	0.59	1.00	1.89	4.80
- Past or current infection	70	0.74	(0.55, 1.01)	0.20	0.40	0.85	1.60	3.40

^1^ Includes all models; sample sizes do not always sum due to missing values. HAV analysis 2001–2006 only.

^2^ Current HBV and recovered HBV groups combined.

### Hepatitis A, B and C status

Of 4,104 women in the hepatitis A model, 1,466 were positive for anti-HAVT, indicating vaccine-derived or natural immunity (Table 3). Of 5,088 women in the hepatitis B model, 40 were positive for anti-HBc and negative for anti-HBs, comprising the current HBV group; 145 were positive for both anti-HBc and anti-HBs, constituting the recovered HBV group; and 1,520 were negative for anti-HBc and positive for anti-HBs, comprising the immunized HBV group. Of 5,071 women in the hepatitis C model, 70 were positive for anti-HCV. The implied prevalence rates are consistent with the epidemiology literature [25].

**Table 3:** Characteristics of study participants by hepatitis serological marker status

	**Hepatitis A**		**Hepatitis B**		**Hepatitis C**	
	**Anti-HAVT **^**1**^		**Anti-HBc **^**2**^		**Anti-HCV **^**3**^	
	**Positive**	**Negative**	**Positive**	**Negative**	**Positive**	**Negative**
N =	1,466	2,638	185	4,903	70	5,001
Age (yrs)	30.5	29.0	36.6	30.5	40.9	30.5
Ethnicity (%)						
- White	22%	51%	22%	41%	51%	40%
- Mexican American	49%	12%	15%	31%	13%	31%
- Other Hispanic	6%	4%				
- Black	17%	30%	48%	24%	30%	25%
- Other	5%	4%	15%	4%	7%	4%
Country born (% US)	51%	94%	61%	76%	96%	78%
Household income (%)						
- < $45,000/yr	63%	48%	72%	52%	85%	52%
- $45,000-$75,000/yr	20%	23%	17%	22%	11%	22%
- > $75,000/yr	17%	29%	12%	26%	5%	26%
Education (%)						
- < HS diploma	45%	28%	29%	33%	38%	33%
- HS diploma	18%	21%	29%	20%	32%	20%
- BA degree	23%	30%	27%	29%	25%	29%
- > BA degree	13%	21%	16%	18%	4%	19%
BMI (% < 30)	68%	67%	64%	67%	66%	67%
Alcohol (% <10 g/d)	89%	87%	84%	87%	70%	87%
Seafood (GM servings)	3.5	3.6	4.0	3.5	3.2	3.6
Anti-HAVT + (%)	--	--	53%	--	48%	--
HBV co-infected (%)	6%	--	--	--	30%	--
HCV co-infected (%)	2%	--	11%	--	--	--

^1^ Anti-HAV positive – current or past infection or immunization; anti-HAV negative - no infection or immunization; HAV analysis 2001–2006 only.

^2^ Anti-HBc positive – current or past (resolved) infection; anti-HBc negative – no infection.

^3^ Anti-HCV positive – current or past infection; anti-HCV negative – no infection.

Women having had HAV, or having been vaccinated for it, did not differ by age, BMI or alcohol intake from those who had not, however, they did differ by socioeconomic parameters (Table 3). Women having come into contact with the hepatitis B virus (including both current and recovered disease) were on average older (by 6 years) than those without HBV contact and more likely to have lower income and lower education attainment, be of Black or Other ethnicity, and be foreign-born. HBV-positive women also ate slightly (10%) more seafood than the sample average, and about 11% were co-infected with HCV. Those positive for HCV were older (by 10 years), and more likely to have lower income and lower education attainment compared to women who were negative for HCV. Unlike those positive for HBV, however, women positive for HCV were more likely to be American-born, to be of mainly White or Black ethnicity, and to consume more alcohol than average. These women ate slightly less (13%) seafood than average, and about 30% were co-infected with HBV.

### Seafood consumption

The GM seafood intake in our eight-year sample of reproductive-age women was 3.56 (95% CI 3.48, 3.65) servings over the month prior to the NHANES interview. The six species most commonly consumed (the greatest number of servings by the largest share of the population) were, in order: shrimp, tuna (all types combined), salmon, crab, catfish, and breaded fish (assumed to be mainly pollock). These species represented 55% of total seafood intake. With the exception of tuna, average mercury tissue concentrations of these species do not generally exceed 0.1 ppm in the US, and are not cautioned against in the national commercial seafood advisory to pregnant and reproductive-age women [39]. Remaining seafood intake was comprised of smaller numbers of women consuming fewer servings spread across 25 other species. The highest-mercury species (e.g., swordfish) were consumed relatively rarely, by few women. Each serving of seafood was associated with an increase in GM TBHg of 6% for the whole sample, an increase of 10% (p < 0.001) when considering women with HBV, and a decrease of 6% (p = 0.28) for those with HCV (results not shown).

### Ratios of blood mercury concentration by hepatitis status

#### HAV Model A

In regression analysis we found no significant association between HAV-positive status and GM TBHg concentrations (Table 4). This did not change after adjusting for estimated MeHg intake, household income, education attainment, ethnicity, BMI, alcohol intake, age, country of birth, and HBV and HCV status (GM TBHg ratio 1.02, 95% CI 0.92, 1.14 p = 0.67).

**Table 4:** Ratio of geometric mean total blood mercury (GM TBHg) concentrations comparing study participants by hepatitis group

		**Unadjusted**			**Adjusted**		
**Model/group**	**N =**	**GM TBHg Ratio **^**1**^	**95% Conf. Int.**	**p- value **^**2**^	**GM TBHg Ratio **^**1**^	**95% Conf. Int.**	**p- value **^**2**^
**Hepatitis A Model **^**3**^ (total n = 4,104)							
- Infection or immunization	1,466	1.01	0.92, 1.11	0.786	1.02	0.92, 1.14	0.674
**Hepatitis B Model **^4^ (total n = 5,088)							
- Current infection	40	1.84	1.37, 2.46	0.000	1.52	1.13, 2.05	0.007
Active	11	4.17	1.90, 8.93	0.000	1.99	0.94, 4.22	0.072
Inactive	29	1.43	1.08, 1.90	0.014	1.40	1.06, 1.88	0.020
- Recovered infection	145	1.31	1.07, 1.59	0.009	1.14	0.93, 1.39	0.205
- Immunized	1,520	1.04	0.96, 1.14	0.270	1.07	0.98, 1.16	0.111
**Hepatitis C Model **^5^ (total n = 5,071)							
- Infection	70	0.72	0.53, 0.98	0.039	0.72	0.51, 0.99	0.049

^1^ Ratio of GM TBHg in the infected (or vaccinated) group compared to the reference group. Ratio of > 1.0 suggests positive and < 1.0 suggests negative association.

^2^ Statistical significance tested using two-tailed t-test, alpha level of 0.05.

^3^ Adjusted for estimated MeHg intake, ethnicity, household income, education attainment, country of birth, alcohol intake, age, body-mass index, HBV and HCV status. HAV analysis 2001–2006 only.

^4^ As for (c), except HBV and HCV status, and including HIV status.

^5^ As for (c), except HCV status.

#### HBV Model

We found a statistically-significant positive association between being in the current HBV group and GM TBHg concentration (Table 4). The association remained after adjusting for estimated MeHg intake, household income, education attainment, ethnicity, country of birth, age, BMI, alcohol intake, and HIV status (GM TBHg ratio 1.52, 95% CI 1.13, 2.05, p < 0.01). The interaction term between seafood intake and chronic hepatitis was statistically significant (p < 0.01). Regression results differed for the two sub-groups with current infection: For the active infection group the positive association after adjustment was stronger (1.99, 95% CI 0.94, 4.22, p = 0.07; n = 11) than for the inactive carrier group (1.40, 95% CI 1.06, 1.88, p = 0.02; n = 29). The association with having recovered from HBV was modestly positive, though not statistically significant (1.14, 95% CI 0.93, 1.39, p = 0.21). We found a weak, non-significant positive association with HBV vaccination (1.07, 95% CI 0.98, 1.16, p = 0.11) after adjusting for the covariates described.

#### HCV Model

We found a modest negative association between GM TBHg concentration and HCV (Table 4). The association remained after adjusting for estimated MeHg intake, household income, education attainment, ethnicity, country of birth, age, BMI, alcohol intake and HBV status, and reached borderline statistical significance (GM TBHg ratio 0.72, 95% CI 0.51, 0.99, p = 0.05).

## Discussion

In this study of a large representative sample of reproductive-age seafood-consuming American women we found those with markers of current chronic HBV infection had 1.52 times the adjusted blood mercury concentration of those never having had contact with the HBV virus or vaccine. The finding was statistically significant thus unlikely due to chance alone. Among those with current disease, the GM TBHg ratio was 1.40 for inactive HBV carriers and 1.99 for those with active HBV, the most severe form of disease in this sample. Women with recovered HBV, the mildest HBV disease state we examined, had 1.14 times the adjusted GM TBHg concentration of the never-infected or vaccinated. Although some strata were small, these results are suggestive of an increasing strength of association with increasing severity of disease which adds weight to our finding. We found no significant association between GM TBHg concentrations and either having (or being vaccinated for) acute-only HAV or having been vaccinated for HBV, situations normally without longer-term risk of damage to the liver or gallbladder. These findings are consistent with our proposed explanation that damage to hepatobiliary elimination structures induced by chronic viral hepatitis could interfere with MeHg’s EH cycle, lead to attenuated MeHg elimination, and be reflected in higher concentrations of circulating TBHg.

The modest negative association (0.72, p = 0.05) we observed between HCV-positive status and GM TBHg concentration was inconsistent with our hypothesis. However, a finding of different outcomes for HBV and HCV is plausible scientifically. While we hypothesized both HBV and HCV would lead to reduced MeHg elimination into bile, the EH cycle can also be accelerated by liver and gallbladder dysfunction reducing uptake into hepatocytes or altering metabolism [18]. For example, MeHg has been found to be eliminated more quickly in laboratory animals with ligated cystic ducts, in which reabsorption to the gallbladder is prevented [16]. Cirrhosis – a more frequent longer-term outcome of HCV than of HBV – has been shown to lead to reduced hepatic uptake due to lower perfusion of cells [18]. Presence of gallstones, associated in the epidemiological literature with HCV but not HBV [30,31,44,45], has also been associated with faster gallbladder emptying [46]. In a recent study, flavonoligands of silymarin – which are eliminated through an EH cycle in circumstances of normal liver health – were found not to undergo EH cycling in HCV patients, whereas substantial EH cycling was found in non-alcoholic fatty liver disease patients [47]. The authors hypothesized HCV-specific changes in hepatobiliary function led to the observed absence of EH cycling. Our HCV findings of lower adjusted MeHg levels among HCV-infected women appear consistent with this research.

To further assess possible reasons for the differing findings for HBV and HCV we also examined six clinical markers of liver function analyzed in the laboratory component of NHANES: alanine transaminase (ALT), aspartate transaminase (ALT), alkaline phosphatase (ALP), gamma glutamyl transpeptidase (GGT), bilirubin and albumin. In our sample, women with current HBV had statistically significant higher ALP and lower albumin levels than those without HBV (Table 5). ALP is observed to be higher in cholestatic conditions where normal bile flow is blocked or interrupted [48]. Those with HCV had statistically significant higher transaminase, ALP and GGT levels. Lower hepatic GGT (which may lead to higher serum GGT) has been associated with faster MeHg elimination through the EH cycle in laboratory animals [16]. In our sample, the magnitude of the negative association between GM TBHg concentration and presence of anti-HCV was strongest in those with the highest serum GGT levels. These liver marker data are suggestive of possible differences in biliary elimination patterns between the HBV- and HCV-infected in our sample. Of additional relevance to our findings, alcohol intake has also been associated with lower blood Hg concentrations [15,49]. Women positive for HCV in our sample had higher than average alcohol intake (whereas those positive for HBV did not), suggesting a further possible factor in the negative association observed.

**Table 5:** Liver enzymes (mean) measured in blood, by hepatitis group

	**ALT**	**AST**	**ALP**	**GGT**	**Bilirubin**	**Albumin**
**Units**	**IU/L**	**IU/L**	**IU/L**	**IU/L**	**mg/dL**	**g/dL**
**Hepatitis A**						
No infection or immunization	19.3	21.4	65.9	18.5	0.65	4.07
Infection or immunization	20.8	21.9	75.7	18.7	0.63	4.07
**Hepatitis B**						
No infection or immunization	20.2	22.0	66.7	19.4	0.64	4.08
Current infection	24.2	24.2	72.4	24.1	0.62	3.92
- Active	22.3	24.5	88.0	21.0	0.65	3.71
- Inactive	24.8	24.0	66.6	25.2	0.61	3.99
Recovered infection	20.2	22.1	65.3	21.0	0.61	4.02
Immunization	18.9	21.6	65.3	16.4	0.67	4.14
**Hepatitis C**						
No infection	20.0	21.7	66.9	19.1	0.64	4.08
Past or present infection	39.2	37.6	70.3	48.9	0.61	3.98

This study had several limitations. Its cross-sectional design precludes inferences regarding causality. Mercury is known to be hepatotoxic in high doses [1], and this could explain the positive association found with HBV infection. Studies in rats and seals have found positive associations between Hg tissue concentrations and levels of some liver enzymes, and have elucidated oxidative stress and other mechanisms of mercury’s hepatotoxicity [20,50]. In humans, clinical case reports have found acute auto-immune hepatitis in association with inorganic Hg overdose [51,52]. With regard to MeHg, elevated standard mortality ratios were found for liver conditions among male Minamata disease survivors in some studies [53,54], but not in others [55]. A positive association between ALT levels and TBHg was observed in a study of US adults, although causality could not be determined due to the cross-sectional design [56]. As noted by several researchers, despite MeHg’s extensive EH cycling and presence in the liver, the scientific literature on its hepatotoxicity is limited [20,50].

While we believe viral hepatitis-induced gallbladder damage and ensuing alteration of MeHg’s EH cycle is the more plausible explanation for both our HBV and HCV findings, other factors we were unable to test may contribute to explaining observed associations. For example, MeHg is eliminated from the body as GSH-conjugates, and GSH depletion is known to reduce the pace of MeHg elimination [15]. Several genetic polymorphisms for production of glutathione-s-transferase (GST) have been associated with MeHg retention [7]. NHANES data for 2001–2008 did not provide data on serum levels of GSH or GST genetic variants, therefore these variables could not be tested in the analysis. It is possible that mercury intake from other sources (e.g., elemental mercury from dental amalgams) may confound our analysis, since other Hg forms can be reflected in TBHg measures [15]. Differences in MeHg toxicokinetics below steady state intake can be important [13] and may play a role in our findings in this US population with comparatively low MeHg exposures. Data were not available to allow us to control for other forms of Hg intake, however there is no *a priori* evidence that women with chronic hepatitis would have higher exposure to other Hg sources than those without. Additional toxicokinetic factors may be involved. For example, presence of chronic HBV may trigger release of stored MeHg in the liver thereby increasing its circulation in blood, as has been hypothesized for tea consumption [57].

It is also possible that Hg’s immunotoxic effects could make exposed individuals vulnerable to viral hepatitis. Animal studies have found chronic exposure to low doses of MeHg initially depress immune function then stimulate an auto-immune response [58]. Studies in a human population in Brazil’s Amazon Basin found that Hg immunotoxicity may be a factor in malaria infection [59]. Neither HBV nor HCV is cytotoxic; liver damage is thought to occur due to the response mounted by the immune system [24,60]. The relationship between Hg immunotoxicity and HBV in particular may warrant further study.

Because of the potential for HBV reactivation, and the blurred line between low-grade infection and recovering disease [24], the inactive HBV carrier category in this analysis may include some women with active infection. In addition, HCV-RNA – a better indicator of chronic HCV – was available for only a portion of our sample, resulting in strata size too small for analysis. However, we believe any possible disease misclassification is likely to have been minor, and would not have had a significant effect on results.

The fact that MeHg intake was estimated based on seafood frequency and species data from a self-report questionnaire is a drawback of study design. This limitation is mitigated to some extent by the careful NHANES survey design and implementation and use of quality control measures described, and by the finding that seafood is among foods least prone to recall bias [34]. The use of TBHg, a biomarker reflective of recent MeHg exposure [15], is consistent with the one-month seafood intake data available in NHANES. However, MeHg’s half-life is on average closer to two months. To address seafood data shortcomings, we performed several sensitivity analyses. In light of higher reported seafood and estimated MeHg intake in the current HBV group than sample average, we ran our model excluding the three observations in this group with highest estimated MeHg intake. The current HBV group’s estimated MeHg intake fell to near sample average and the adjusted GM TBHg ratio was 1.40 (95% CI 1.07, 1.83, p = 0.02), broadly consistent with our main HBV model findings (results not shown). We also evaluated intake of the most commonly-consumed seafood species and found patterns across disease categories were not markedly different.

## Conclusions

This study found that TBHg levels varied by hepatitis status among American reproductive-age seafood-consuming women, and the observed associations remained after adjusting for estimated MeHg intake from seafood consumption and other predictors. While limited by the constraints of a cross-sectional design, these results raise the possibility that chronic viral liver disease may alter the pace of MeHg hepatobiliary elimination in reproductive-age women – and therefore also affect the risk of neurotoxicity to the developing fetus. The finding that seafood-consuming reproductive-age women with chronic hepatitis B had 1.52 times the blood mercury concentrations as women having had no contact with the virus is of public health relevance. In particular, it suggests offspring of HBV-positive high seafood-consuming women may be at higher risk of verbal, spatial and fine-motor skill delays associated with MeHg-induced developmental neurotoxicity. The implications may be particularly relevant in populations where chronic HBV prevalence and seafood intake are both high (such as US women of Asian and Pacific Islands ethnicity [24,61]).

This representative US sample was ethnically and socially heterogenous, therefore it is reasonable to consider possible implications of generalizing the findings internationally. Higher TBHg levels are observed in a number of regions – e.g., Southeast Asia, the Amazon Basin, and the Arctic [3] – where HBV is also endemic [62,63]. Further study of the impact of liver disease on MeHg elimination is warranted, in both animals and humans. Possible policy implications include enhancing risk-communication through targeting of commercial seafood advisories, adding mercury testing to existing HBV screening programs for reproductive-age women, and enhanced high-risk population surveillance.

## Abbreviations

Anti-HAVT: Total hepatitis A antibody; Anti-HBc: Hepatitis B core antibody; Anti-HBs: Hepatitis B surface antibody; Anti-HCV: Hepatitis C antibody; EH: Enterohepatic; ELISA: Enzyme-linked immunosorbent assay; GM: Geometric mean; HBsAg: Hepatitis B surface antigen; Hg: Mercury; MeHg: Methylmercury; TBHg: Total blood mercury.

## Competing interests

All authors declare they have no completing financial or non-financial interests.

## Authors’ contributions

MAF participated in the design of the study, statistical analysis and interpretation of findings. TAB, PNB and ANA participated in the design of the study and interpretation of findings. JM participated in the statistical analysis. MCS conceived of the study, participated in study design, statistical analysis and interpretation of findings and drafted the manuscript. All authors read and approved the final manuscript.
